# Chorioretinal thickness and retinal pigment epithelial degeneration of fellow eyes in patients with unilateral neovascular age-related macular degeneration with subretinal drusenoid deposits

**DOI:** 10.1186/s12886-022-02518-4

**Published:** 2022-07-14

**Authors:** Dongwan Kang, Eun Gyu Yoon, Ki Tae Nam, Cheolmin Yun

**Affiliations:** Department of Ophthalmology, Korea University College of Medicine, Seoul, Korea

**Keywords:** Age-related macular degeneration, Neovascularization, Subretinal drusenoid deposit, Retinal pigment epithelium

## Abstract

**Background:**

We sought to investigate the chorioretinal thickness and retinal pigment epithelial (RPE) degenerative features of eyes with early age-related macular degeneration (AMD) and subretinal drusenoid deposits (SDDs) according to the presence of macular neovascularization (MNV) in the fellow eyes.

**Methods:**

We classified 70 eyes into two groups of 47 eyes with non-neovascular AMD and 23 eyes with neovascular AMD, respectively, according to the presence of MNV in the fellow eyes. The mean macular retinal, ganglion cell–inner plexiform layer (GCIPL), and choroidal thickness values and RPE features of the 6-mm-diameter zone were compared. RPE degeneration was defined as a lesion with an incomplete RPE and outer retinal atrophy (iRORA) or attenuated RPE reflectivity with diffuse basal laminar deposits, which was defined as when the eye showed an attenuated RPE line with granular features and mixed reflectivity in combination with sub-RPE deposits with a lesion ≥ 1,000 µm in length.

**Results:**

Mean retinal, GCIPL, and choroidal thickness values (286.69 ± 15.02 µm, 64.36 ± 4.21 µm, and 156.11 ± 33.10 µm) of the neovascular AMD group were greater than those (278.61 ± 13.96 µm, 61.44 ± 4.63 µm, and 133.59 ± 34.33 µm) of the non-neovascular AMD group (all *P* < 0.05). RPE degeneration was more prevalent in the neovascular AMD group (65.2%) than the non-neovascular AMD group (38.3%; *P* = 0.034). Greater mean GCIPL and choroidal thickness values and the presence of RPE degeneration were associated with type 3 MNV in fellow eyes (all *P* < 0.05).

**Conclusions:**

Different degenerative features according to MNV in fellow eyes of patients with AMD and SDDs suggest that variable degenerative features might be present during disease progression and have an association with the phenotype.

**Supplementary Information:**

The online version contains supplementary material available at 10.1186/s12886-022-02518-4.

## Background

Age-related macular degeneration (AMD) is a disease with degenerative features of the outer retina whose most typical feature of AMD is drusen [1–4]. With the development of imaging techniques, drusen have been classified into several types, and patients’ clinical features also vary according to these types [5–7]. Subretinal drusenoid deposits (SDDs) have a characteristic location, shape, and associated chorioretinal changes relative to those of soft drusen and pachydrusen [5–10]. SDDs are usually observed in the superior or superotemporal outer macular region as interlacing drusen-like deposits, and these were found to be hyper-reflective accumulations above the retinal pigment epithelium (RPE) on optical coherence tomography (OCT) [11, 12]. Eyes with SDDs are characterized by a thin choroid and outer retinal atrophy associated with the regression of SDDs, and the presence of SDDs has been reported to be strongly associated with the development of geographic atrophy (GA) and neovascular AMD, especially type 3 macular neovascularization (MNV) [5–7, 13, 14]. The etiology of SDD formation is still unclear, but it has been suggested to be associated with the dysregulation of physiologic pathways of lipid and retinoid transfer accompanied by diffuse alterations of the choroid, choriocapillaris, and RPE [6, 7, 13, 15–19].

The natural history of early AMD eyes with SDDs has not yet been clearly investigated, but several studies to date have reported that the retina and choroid become thinner and the features of RPE change over time in affected patients, and the process was suggested to be an overall bilateral degeneration [6, 7, 13, 15, 20–22]. With disease progression, late AMD can develop, but the characteristics of the retina, GCIPL, choroid, and RPE in fellow eyes of patients with unilateral neovascular AMD have been insufficiently reported to date. Elucidating the clinical characteristics of fellow eyes may provide information about the forme fruste features of neovascular AMD with SDDs.

In this study, we investigated the characteristics of early to intermediate AMD eyes with SDDs before the stage of geographic atrophy according to the presence of MNV in another eye. In addition, we tried to find some other forme fruste characteristics of MNV considering the degenerative features of the RPE and chorioretinal thickness and supposed that this might present in patients with neovascularization in one eye based on the bilateral degenerative characteristics of AMD patients.

## Methods

This study was conducted after receiving approval from the institutional review board of Korea University Medical Center and following the tenets of the Declaration of Helsinki.

We retrospectively reviewed the medical records of consecutive patients with AMD and SDDs diagnosed between November 2016 and November 2020 at Korea University Medical Center. All patients underwent a comprehensive ophthalmic examination, including fundus photography, autofluorescence imaging, fluorescein angiography, indocyanine angiography, and optical coherence tomography (OCT). We classified eyes as those with early AMD according to the Age-related Eye Disease Study (AREDS) grading system [1]. Soft drusen were considered to be present if yellowish round to ovoid deposits measuring >63 µm in diameter with poorly defined borders were aggregated in the macula on fundus photography and showed sub-RPE accumulation with homogenous reflectivity on OCT images [6, 23]. We included patients with early AMD in at least one. If the patient had late AMD (neovascular AMD or geographic atrophy) in both eyes, they were excluded, and if the patient had one eye with neovascular AMD, the fellow eye with early AMD was chosen for analysis. Eyes with a history of retinal disease, including retinal vein occlusion, diabetic retinopathy, or epiretinal membrane at the macula; high myopia with an axial length of ≥26.0 mm (or the spherical equivalent < −6.0 diopters); past vitreoretinal surgery, intravitreal injection, or retinal laser treatment; or glaucoma were excluded. In addition, age-matched controls without a history of vitreoretinal disease were selected from the OCT database.

OCT and fundus autofluorescence images were acquired with the Spectralis HRA system (Heidelberg Engineering, Heidelberg, Germany). Macular OCT images were acquired using the volume scan with a 30- × 25-degree area centered on the fovea (61 horizontal B-scans, 120-µm interscan distance, average of 30 automatic real-time tracking frames). Fundus autofluorescence images were obtained with a wavelength of 488 nm and a 30-degree field of view.

SDDs were diagnosed if the eye showed ≥5 hyper-reflective, triangular-shaped subretinal lesions above the RPE on a single OCT B-scan within a 6-mm area according to the Early Treatment Diabetic Retinopathy Study (ETDRS) grid [24]. GA was defined if the eye presented a lesion exhibiting decreased signal intensity on fundus autofluorescence imaging with a diameter of >175 µm and complete RPE and outer retinal atrophy on OCT imaging [25]. If the region showed a zone of attenuation or disruption of the RPE accompanied by hypertransmission into the choroid, it was classified as RPE atrophy, while if the region showed loss of the ellipsoid zone, interdigitation zone, and external limiting membrane and outer nuclear layer thinning, it was categorized as outer retinal atrophy. Classification of MNV types was based on multimodal imaging, including fluorescein angiography, indocyanine green angiography, and OCT [26].

The mean retinal, GCIPL, and choroidal thickness values of the 6-mm zone per the ETDRS chart were acquired. The mean retinal thickness was defined as that between the internal limiting membrane (ILM) and Bruch’s membrane. The GCIPL thickness was calculated from the sum of the ganglion cell layer (GCL) thickness and inner plexiform layer (IPL) thickness. Segmentation errors of the B-scans were reviewed and, if present on those images of eyes with ILM, the inner border of the GCL, outer border of the IPL, and Bruch’s membrane were adjusted manually. The choroidal thickness was defined as the vertical distance from Bruch’s membrane to the chorioscleral interface and was measured manually. The mean choroidal thickness was calculated from the mean of the values collected from nine points (i.e., fovea, 750 µm, 1,500 µm, 2,250 µm, and 3,000 µm both nasally and temporally from the fovea) using the horizontal line scanning with an enhanced-depth imaging option.

**Fig 1.**
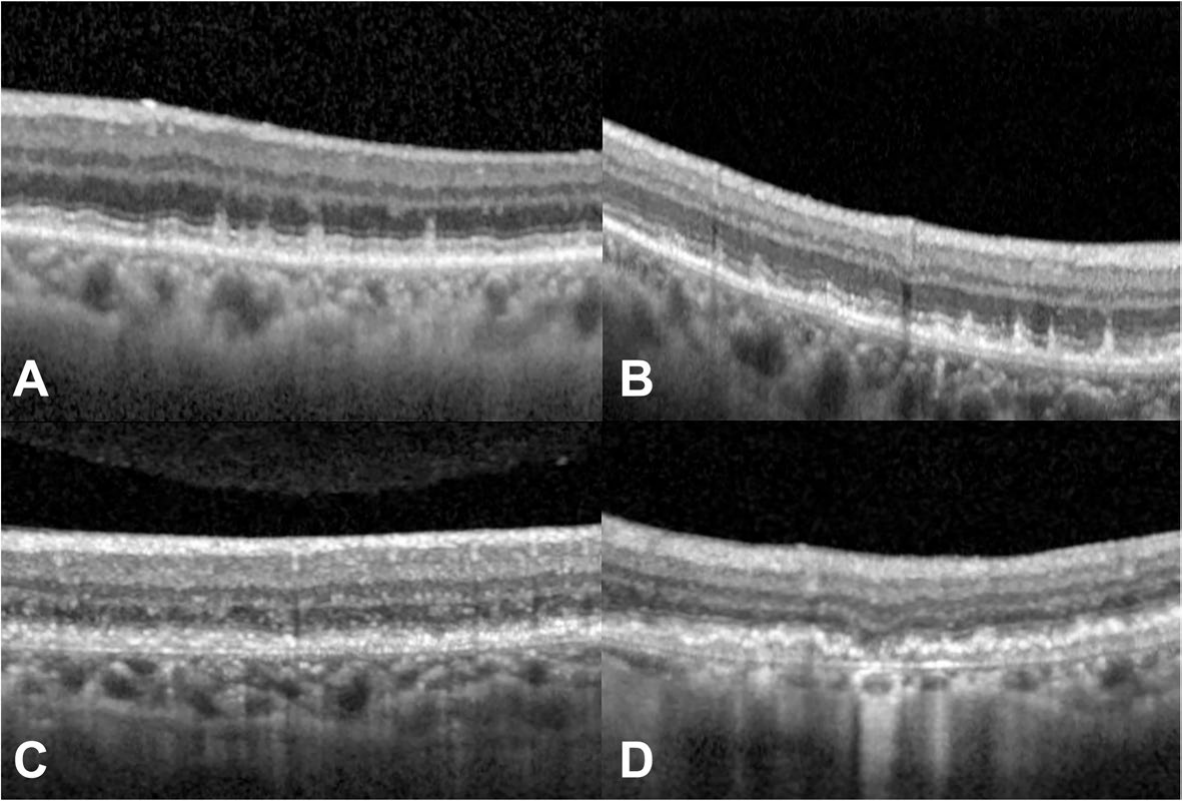
Classification of the retinal pigment epithelium (RPE) and Bruch’s membrane complex based on optical coherence tomography. (**A**) Only subretinal drusenoid deposits (SDDs); (**B**) SDD and sub-RPE deposits; (**C**) SDD, sub-RPE deposits with attenuated RPE, and outer retinal atrophy; and (**D**) SDD, sub-RPE deposits with incomplete RPE, and outer retinal atrophy.

Two investigators (D. K. and K. N.) classified the features of the RPE–Bruch’s membrane complex within the 6-mm zone of the ETDRS chart and classified them into the following four types: only SDDs, SDDs and sub-RPE deposits, sub-RPE deposits with attenuated RPE, and sub-RPE deposits with incomplete RPE and outer retinal atrophy (iRORA) (Figure 1) [20, 25]. Eyes with only SDDs showed no signs of visible sub-RPE deposits, and the RPE–Bruch’s membrane complex looked to be a single structure. Eyes with SDD and sub-RPE deposits were defined when the eyes showed a double layer consisting of lines of RPE and Bruch’s membrane, separated by sub-RPE deposits measuring ≥1,000 µm in length and seen on ≥3 consecutive B-scans. Eyes with SDD and sub-RPE deposits with attenuated RPE were defined when the eyes showed an attenuated RPE line with granular features and mixed reflectivity in combination with sub-RPE deposits with a lesion size of ≥1,000 µm in length and seen on ≥3 consecutive B-scans with or without outer retinal atrophy. Eyes with SDD and sub-RPE deposits with iRORA were defined if the eye showed disruption of the RPE band of <250 µm in length accompanied by outer retinal atrophy [25]. The eyes with sub-RPE deposits with attenuated RPE or sub-RPE deposits with iRORA were included into the RPE degeneration group, while other cases were classified into an RPE non-degeneration group. If discordance between the two investigators regarding a classification occurred, a third reviewer (C. Y.) reviewed the case in question and made the final call.

In each patient, only one eye was chosen for analysis, considering the possible bias that might occur from the biological correlation between two eyes of a single patient. If the early AMD in both eyes was considerable, the right eye was chosen and, if the right eye did not meet the inclusion and exclusion criteria, the left eye was selected. Intergrader reliability for the RPE features was assessed with the kappa coefficient. Statistical analysis was performed using the Statistical Package for the Social Sciences version 20.0 for Windows software program (IBM Corporation, Armonk, NY, USA). *P* < 0.05 was considered to be statistically significant.

## Results

A total of 70 eyes with early AMD with SDDs of 70 patients and age-matched 47 control eyes were included. The AMD eyes were classified as 47 eyes with non-neovascular AMD and 23 eyes with neovascular AMD according to the existence of neovascular AMD in fellow eyes, respectively. Baseline characteristics were not different among the groups (Table 1).

**Table 1.** Baseline characteristics of the eyes among groups

	SDD group		Control group (*n* = 47)	*P* value
	Non-neovascular AMD group (*n* = 47)	Neovascular AMD group (*n *= 23)		
Age (years)	76.26 ± 5.53	75.83 ± 4.53	74.91 ± 5.24	0.318*
Sex (male:female)	16:31	8:15	23 : 24	0.284^†^
Axial length (mm)	22.76 ± 1.18	23.05 ± 1.33	22.84 ± 1.00	0.609*
IOP (mmHg)	15.38 ± 1.97	15.09 ± 1.81	15.53 ± 1.98	0.668*
Hypertension (n, %)	24 (51.1%)	9 (39.1%)	21 (44.7%)	0.621^†^
Diabetes (n, %)	13 (27.7%)	7 (30.4%)	10 (21.3%)	0.655^†^

*SDD* Subretinal drusenoid deposit, *AMD* Age-related macular degeneration, *GCIPL* Ganglion cell–inner plexiform layer, *CT* Choroidal thickness, *RPE* Retinal pigment epithelium

^*^P value is based on the analysis of variance test

^†^*P* value is based on the Chi-square test

**Fig 2.**
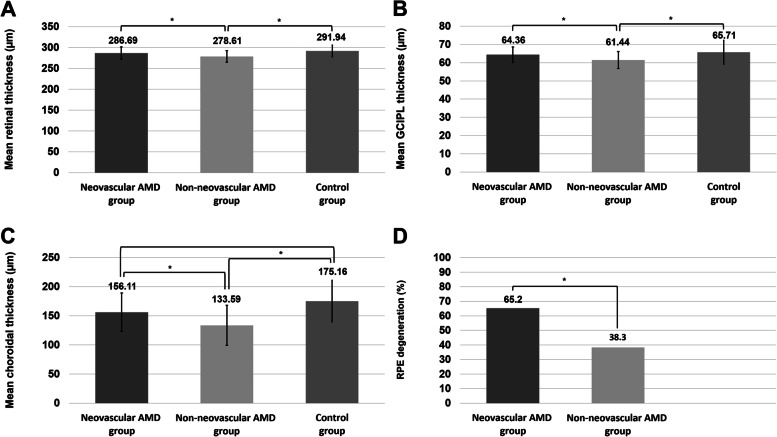
Comparison of retinal, ganglion cell–inner plexiform (GCIPL) layer, and choroidal thickness values and the rate of retinal pigment epithelium (RPE) degeneration among eyes with early age-related macular degeneration (AMD) with subretinal drusenoid deposits (SDDs) according to the presence of neovascularization in another eye and normal control group. The mean retinal thickness (**A**), GCIPL thickness (**B**), choroidal thickness (**C**), and rate of RPE degeneration (**D**) of the neovascular AMD group (having another eye with neovascular AMD) were greater than those of the non-neovascular AMD group (having both eyes with early AMD and no neovascular AMD in any eye). The mean retinal and GCIPL thickness values of the neovascular AMD group were similar to those of the normal control group. The *P* value was determined based on the analysis of variance test. An asterisk (*) indicates that the values differ significantly from one another according to the post-hoc analysis with Duncan’s test.

The mean retinal, GCIPL, and choroidal thickness values of the neovascular AMD group (286.69 ± 15.02 µm, 64.36 ± 4.21 µm, and 156.11 ± 33.10 µm), control group (291.94 ± 14.05 µm, 65.71 ± 6.56 µm, and 175.16 ± 35.67 µm), and non-neovascular AMD group (278.61 ± 13.96 µm, 61.44 ± 4.63 µm, and 133.59 ± 34.33 µm) were different (analysis of variance [ANOVA] test, *P* < 0.001, *P* = 0.001, and *P* < 0.001, respectively) (Figure 2). Post-hoc analysis revealed that the mean retinal thickness, GCIPL thickness, and choroidal thickness of the neovascular AMD group and control group were greater than those of the non-neovascular AMD group (Supplementary Table 1). RPE degeneration was more common in the neovascular AMD group (65.2%) than in the non-neovascular AMD group (38.3%) (*P* = 0.034).

**Fig 3.**
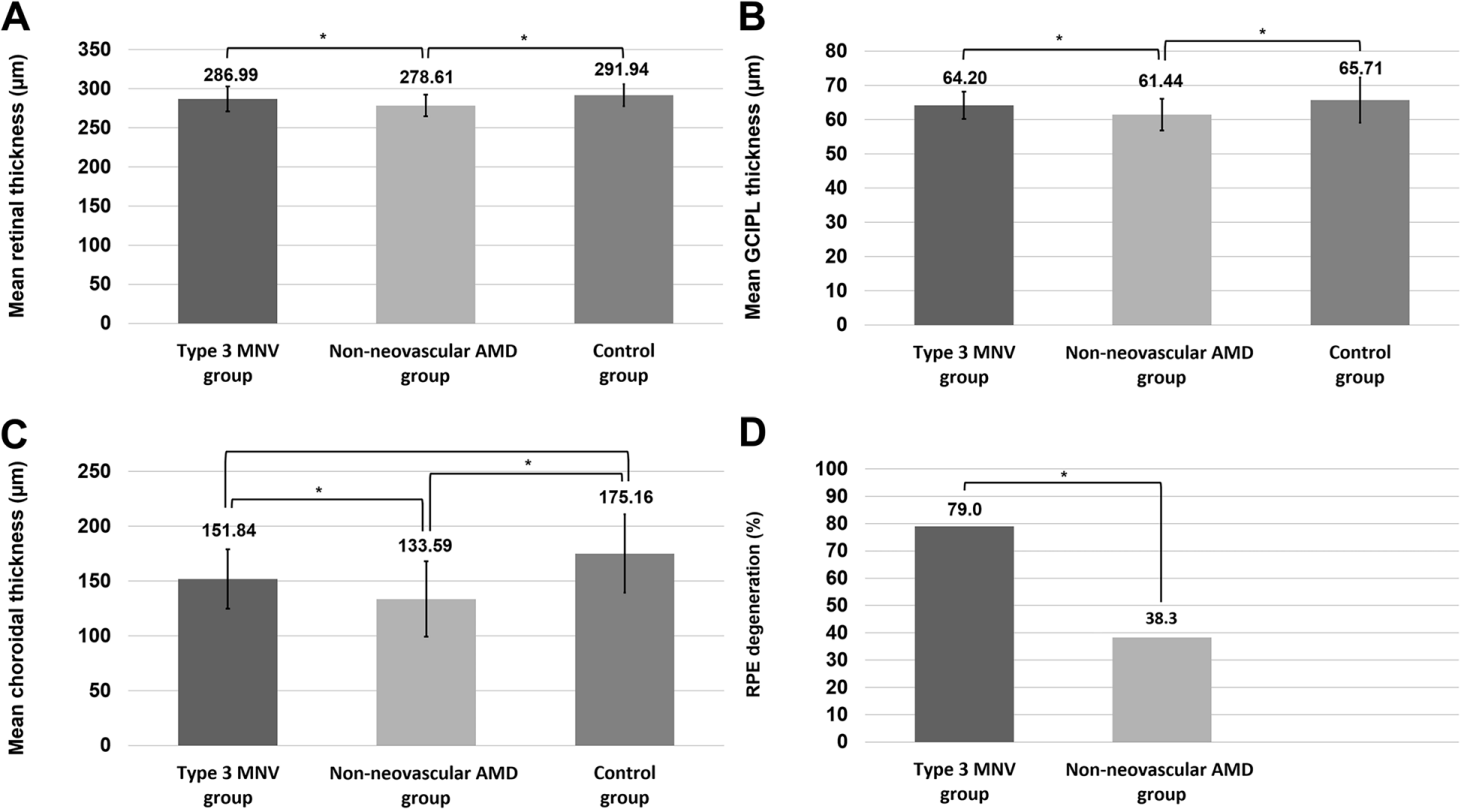
Comparison of retinal, ganglion cell–inner plexiform (GCIPL) layer, and choroidal thickness values and the rate of retinal pigment epithelium (RPE) degeneration among early eyes with age-related macular degeneration (AMD) with subretinal drusenoid deposits (SDDs) according to the presence of type 3 macular neovascularization (MNV) in another eye and the normal control group. The mean retinal thickness (**A**), GCIPL thickness (**B**), choroidal thickness (**C**), and rate of RPE degeneration (**D**) of the type 3 MNV group (having another eye with type 3 MNV) were greater than those of the non-neovascular AMD group (having both eyes with early AMD and no neovascular AMD in any eye). The mean retinal and GCIPL thickness values of the type 3 MNV group were similar to those of the normal control group. *P*-value was based on the analysis of variance test. An asterisk (*) indicates that the values differ significantly from one another according to the post-hoc analysis with Duncan’s test.

**Fig 4.**
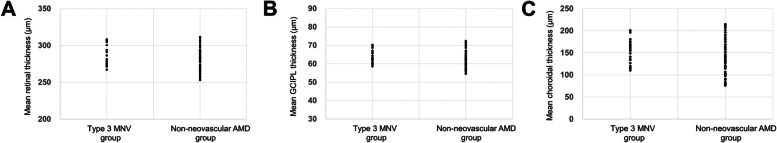
Distribution of the mean retinal (**A**), ganglion cell–inner plexiform layer (GCIPL) (**B**), and choroidal thickness (**C**) values of the type 3 MNV group (having another eye with type 3 MNV) and the non-neovascular AMD group (having both eyes with early AMD and no neovascular AMD in any eye). The retinal, GCIPL, and choroidal thickness values of the non-neovascular AMD group are distributed more widely than those of the type 3 MNV group, and the type 3 MNV group shows relatively highly distributed thickness values compared to those of the non-neovascular AMD group.

Among the 23 eyes of the neovascular AMD group, 1 eye (4.3%), 3 eyes (13.0%), and 19 eyes (82.6%) were classified into type 1, 2, and 3 MNV, and no eyes presented polypoidal choroidal vasculopathy. Mean retinal, GCIPL, and choroidal thickness values of fellow eyes in the type 3 neovascular AMD subgroup (286.99 ± 15.96 µm, 64.20 ± 4.01 µm, and 151.84 ± 27.08 µm), control group, and non-neovascular AMD group were also different (ANCOVA test, *P* < 0.001, *P* = 0.001, and *P* < 0.001, respectively) (Figures 3 and 4). Post-hoc analysis revealed that the mean retinal thickness, GCIPL thickness, and choroidal thickness of the type 3 neovascular AMD subgroup and control group were greater than those of the non-neovascular AMD group (Supplementary Table 2). RPE degeneration was more common in the neovascular AMD group (79.0%) than in the non-neovascular AMD group (38.3%) (*P* = 0.003).

**Fig 5.**
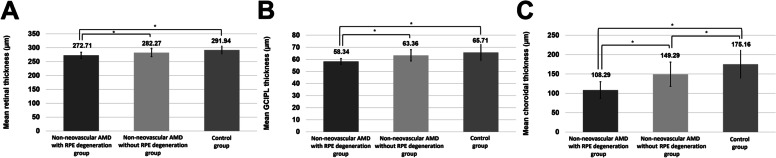
Comparison of retinal, ganglion cell–inner plexiform (GCIPL) layer, and choroidal thickness values of eyes with non-neovascular AMD (having both eyes with early AMD and no neovascular AMD in any eye) according to the retinal pigment epithelium (RPE) degeneration. According to this subgroup analysis, the non-neovascular AMD group with RPE degeneration had lower retinal, GCIPL, and choroidal thickness values than those of the non-neovascular AMD group without RPE degeneration and the normal control group. *P*-value was based on the analysis of variance test. An asterisk (*) indicates that the values differ significantly from one another according to the post-hoc analysis with Duncan’s test.

In the non-neovascular AMD group, 29 eyes and 18 eyes were classified into subgroups without RPE degeneration and with RPE degeneration, respectively. The mean retinal, GCIPL, and choroidal thickness values of the subgroup with non-neovascular AMD without RPE degeneration (282.27 ± 14.43 µm, 63.36 ± 4.67 µm, and 149.29 ± 31.39 µm) and control group were greater than those of the subgroup with non-neovascular AMD with RPE degeneration (272.71 ± 11.18 µm, 58.34 ± 2.36 µm, 108.29 ± 21.66 µm) (ANOVA test, *P* = 0.021, *P* < 0.001, and *P* < 0.001, respectively) (Figure 5).

**Fig 6.**
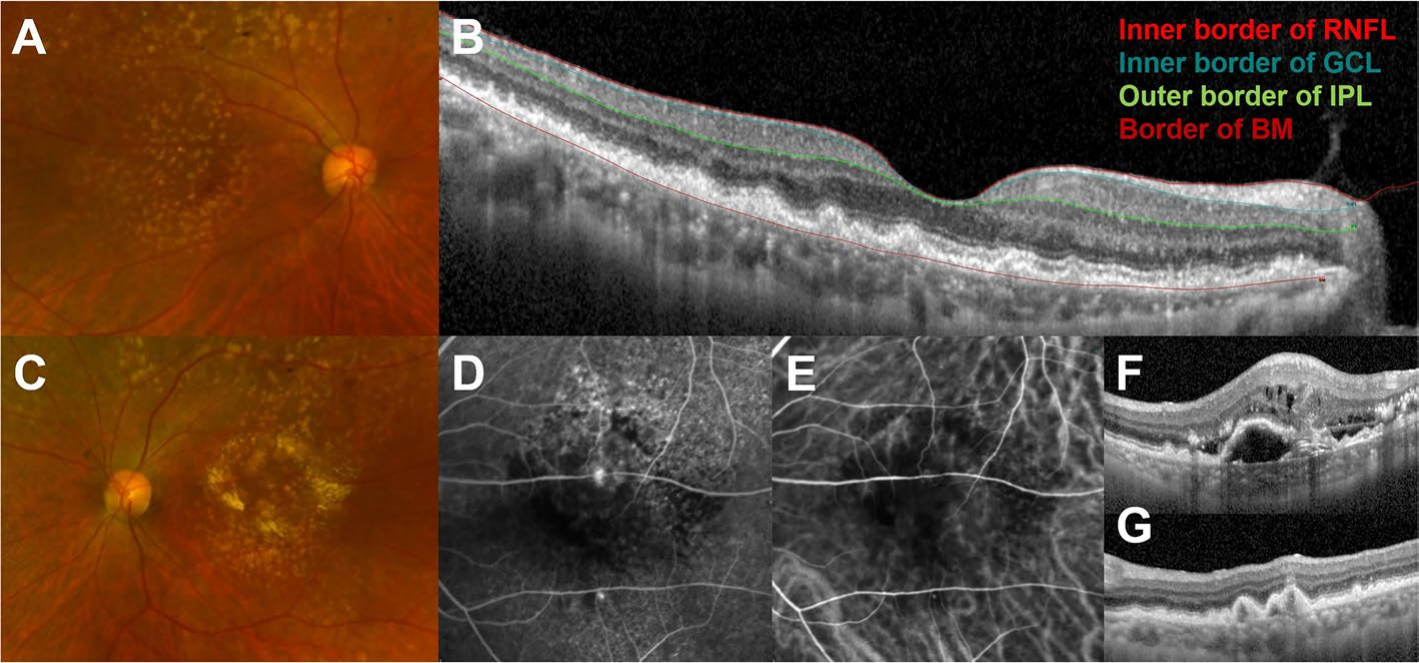
A representative case of a 75-year-old female patient with dry AMD in the right eye and type 3 macular neovascularization in the left eye. A fundus photo (**A**) shows numerous soft drusen, and an optical coherence tomography image (**B**) shows semicircular soft drusen and subretinal drusenoid deposits in the right eye. The inner border of the retinal nerve fiber layer (red color), inner border of the ganglion cell layer (dark green color), outer border of the inner plexiform layer (blue color), and border of Bruch’s membrane (dark red color) are presented. A fundus photo of left eye (**C**) also shows numerous soft drusen, and fluorescein angiography (**D**) and indocyanine angiography (**E**) reveals two hotspots suggesting chorioretinal anastomosis. OCT images corresponding to hot spots show pigment epithelial detachment with intraretinal and subretinal fluid (**F**, upper hot spot) and drusenoid pigment epithelial detachment with intraretinal hyperreflectivity (**G**, lower hot spot).

Logistic regression analysis revealed that type 3 neovascularization in 1 eye of the patients was associated with the non-neovascular fellow eye’s mean retinal, GCIPL, and choroidal thickness values and the presence of RPE degeneration, respectively (Table 2). Multivariate analysis revealed that the non-neovascular AMD eye’s mean GCIPL and choroidal thickness values and the presence of RPE degeneration were associated with type 3 MNV in the other eye (Table 3). The kappa coefficient for intergrader reliability of the RPE features was 0.642. A representative case is presented in Figure 6.

**Table 2.** Univariate analysis for estimating the risk factors of type 3 macular neovascularization in 1 eye among the patients with dry age-related macular degeneration with subretinal drusenoid deposits based on the parameters of non-neovascular AMD eyes

Variables	*P* value*	OR (95% CI)
Age (years)	0.761	1.016 (0.917–1.125)
Sex (male)	0.073	0.342 (0.106–1.103)
History of hypertension	0.178	0.471 (0.158–1.409)
History of diabetes	0.134	2.380 (0.766–7.396)
Axial length (mm)	0.580	1.137 (0.722–1.791)
Intraocular pressure (mmHg)	0.853	1.029 (0.763–1.387)
Mean retinal thickness (µm)	0.044	1.040 (1.001–1.080)
Mean GCIPL thickness (µm)	0.032	1.145 (1.012–1.295)
Mean choroidal thickness (µm)	0.049	1.018 (1.000–1.035)
RPE degeneration	0.005	6.042 (1.731–21.086)

*GCIPL* Ganglion cell–inner plexiform layer, *RPE* Retinal pigment epithelium, *OR* Odds ratio, *CI* confidence interval

**Table 3.** Multivariate analysis for estimating the risk factors of type 3 macular neovascularization in 1 eye among the patients with dry age-related macular degeneration with subretinal drusenoid deposits based on the parameters of non-neovascular AMD eyes

Variables	*P* value*	OR (95% CI)
Mean GCIPL thickness (µm)	0.019	1.218 (1.033–1.435)
Mean choroidal thickness (µm)	0.024	1.027 (1.003–1.051)
RPE degeneration	<0.001	21.304 (3.814–118.998)

*GCIPL* Ganglion cell–inner plexiform layer, *RPE*, Retinal pigment epithelium, *OR* Odds ratio, *CI* Confidence interval

## Discussion

In this study, eyes with early AMD and SDDs showed different thickness and features of the retina, choroid, and RPE according to the presence of neovascular AMD and type 3 MNV. Fellow eyes with neovascular AMD showed greater proportions of RPE degeneration and a thicker retina and choroid. Considering that the retina and choroid were thinner in eyes with RPE degeneration than in those without RPE degeneration among non-neovascular AMD patients, the fellow eyes of patients with neovascular AMD might experience more prominent degeneration in the RPE–Bruch’s membrane complex with a relatively preserved retina.

Several studies have reported on the characteristic features of the GCIPL, retina, choroid, and RPE in eyes with dry AMD and SDDs [7, 13–15, 20, 27]. Arising from choroidal insufficiency, the blood flow to the choriocapillaris can be impaired, which may lead to subsequent changes in the RPE, outer retina, and inner retina [7, 13, 14, 27]. The natural history of changes in the eyes has not been clearly elucidated, but several studies previously assumed that these eyes are experiencing an overall degenerative process [7, 13, 14, 27].

Persistent integrity of the RPE is important for appropriate functioning of the outer retina in eyes with AMD [4, 28–30]. Previous studies have reported features of the RPE in eyes with dry AMD and RPE degeneration has been known to be a key feature of eyes with dry AMD and SDDs [4, 20, 21, 28–30]; however, because of the lack of long-term follow-up investigations of eyes with SDDs, the natural history of RPE features in these eyes is still not clearly defined. Several studies have reported progressive thinning of the choroid and retina with time and suggested that early AMD eyes with SDDs are prone to overall chorioretinal degeneration, including of the RPE [20, 22, 27]. In addition, basal laminar deposits are a key marker for AMD and, with advances in the disease, thicker basal laminar deposits are correlated with severe degeneration of the RPE [31]. In this study, lower retinal and choroidal thickness values in eyes with RPE degeneration compared to in those without may indicate that these eyes experience degeneration of the retina and choroid accompanied by RPE degeneration, and deposition of basal laminar deposits and associated RPE changes might be a sign of late-stage disease [20, 31].

GA is an end status of dry AMD, and affected eyes show complete loss of the RPE and outer retina [14, 29]. The development of neovascular AMD is associated with a breakdown of the outer retinal barrier consisting of the RPE and Bruch’s membrane in the focal environment [2, 4, 26, 32]. The exact pathomechanism of neovascularization remains unclear, but several theories have been suggested [2, 4, 6, 26, 32]. Sarks et al. showed a pathway for drusen-related type 1 MNV, and this was suggested to be contributed to by pro-inflammatory and pro-angiogenic lipids in drusen material [16, 17]. Based on the previous reports, many steps between drusen and type 1 MNV are visible; however, this is not the case for type 3 MNV. With the deposition of sub-RPE materials, the environment for the outer retina and RPE is changed and subsequent RPE and outer retina abnormalities can develop [3, 31]. In addition to sub-RPE deposits, the RPE in eyes with SDDs is implicated to be under hypoxia from the choroid and choriocapillaris insufficiency, and the hypoxic environment of the outer retina may cause increased vascular endothelial growth factor (VEGF), migration of RPE cells, which are presented as hyper-reflective foci on OCT, and subsequent neovascularization [13, 32, 33]. In these circumstances, an imbalance between pro-angiogenic and anti-angiogenic growth factors in the outer retina may lead to the formation of neovascularization [2, 26, 32]. Higher VEGF levels in the retina have been suggested to partially stem from degenerative RPE cells and may promote intraretinal neovascularization with detached and degenerative RPE [32]. Furthermore, significantly heightened intraocular VEGF levels in eyes with type 3 neovascularization relative to other types of neovascularization may implicate diffuse hypoxic conditions in these eyes [34]. In addition to these generalized hypoxic conditions, an imbalance between pro-angiogenic and anti-angiogenic factors in the focal area caused by discrepancies in metabolic demand between the outer retina and choriocapillaris might lead to neovascularization [32]. If the degeneration process is relatively slow, RPE atrophy may keep in step with subsequent retinal atrophy [20, 27, 35]. During this process, decreased metabolic demand in the retina might not stimulate the production of pro-angiogenic growth factors, and a steady synchronized overall retinal atrophy might lead to GA [27].

Eyes with dry AMD with SDD can experience the degenerative process associated with both soft drusen and SDDs [23, 27]. Retinal, choroidal, and RPE degeneration can occur with drusen- or SDD-associated degenerative process, and the degenerative features might depend on which type of degenerative process is prominent. Thus, various degenerative features might present in these eyes. As an aspect of the non-neovascular AMD group which didn’t have any MNV in both eyes in this study, eyes might present the various phase of degeneration from the early phase of AMD to the phase prior to the development of GA. The thinner retina and choroid in the non-neovascular AMD group with RPE degeneration compared to those of the group without RPE degeneration may suggest that the eyes without MNV development are experiencing a synchronized overall retinal, RPE, and choroidal thinning. However, in the neovascular AMD group, the RPE degeneration was more prominent than the retina and choroid changes in this study. This might come from various conditions associated with drusen or basal laminar deposits, which might create an environment prone to prominent RPE degeneration. In some eyes with AMD, the degree of RPE degeneration and the retinal and choroidal degeneration can be different, and the relatively preserved outer retina, which was presented as a thickness in this study, and a prominent degenerated RPE might induce an increase in pro-angiogenic factors, which might be associated with the MNV. However, the magnitude of the differences between groups varied according to the parameters. Differences in mean choroidal thickness and rates of RPE degeneration were relatively greater than those in mean retinal thickness, which showed only about an 8-µm difference. This might come from the fact that notable changes in AMD eyes with SDD mainly occur in the choroid and RPE, and changes in the retina might be secondary to the changes in the choroid and RPE [20–22, 27]. The changes in choroidal thickness and RPE integrity may be more valuable for estimating the status of these eyes than the retinal thickness.

Neovascular AMD can also develop in eyes with pre-existing GA [36, 37]. Eyes with neovascular AMD, especially type 3 neovascularization, may have different clinical features according to the presence of GA, and those eyes with atrophy have a lower disease activity and recurrence rate [36, 38]. This might suggest that type 3 neovascularization that develops earlier (before GA development) versus later (after GA development) might have different characteristics. However, because we excluded eyes with GA and investigated to elucidate the degenerative features of early AMD eyes with SDDs before the development of GA, we cannot suggest the features of fellow eyes that already have GA and neovascularization.

Several studies have reported fellow eye characteristics of AMD with type 3 neovascularization, suggesting they include atrophy of the RPE, the presence of reticular pseudodrusen, increased numbers of drusen, hyper-reflective foci, and reduced retinal and choroidal blood flow [6, 33, 39, 40]. In this study, we classified RPE features concisely to assess the status of eyes with certain retina, GCIPL, and choroidal thickness values intuitively. Multivariate analysis revealed that risk factors for fellow eye neovascularization included increased GCIPL or choroidal thickness values and the presence of RPE degeneration. AMD is a neurodegenerative disease that results in cell death in the outer retina and RPE [41]. Retinal thickness is affected by the AMD status, which has been reported to be primarily due to outer retinal layers [42]. Because the retinal thickness was recorded as the thickness between the inner border of the retinal nerve fiber layer and Bruch’s membrane using the built-in software of the OCT system in this study, the retinal thickness also included the thickness of drusen or sub-RPE deposits in addition to the neurosensory retina. Based on the finding that consequent neuronal loss can develop after the outer retinal degeneration, GCIPL thickness has also been suggested to be a surrogate for retinal degeneration with progression of the disease [43, 44]. We also included the GCIPL thickness and confirmed a clinical significance in this study [27].

This study has several limitations. First, it was of a retrospective nature with a small number of cases. Because this study included the early or intermediate AMD eyes presented with SDDs and excluded eyes without SDDs, the prevalence of type 3 MNV was significantly higher [7, 26, 45]. The results of this study should be interpreted with consideration of selection bias. Second, because this study was a cross-sectional investigation, we could not evaluate the long-term progress of the disease and thus cannot suggest the clinical significance of the results. In addition, we could not provide the model of the progression of drusen to type 1 MNV based on the current form of this study [16, 46]. Third, even though 3 reviewers classified the RPE features, the assessment was subjective and the lack of an objective method might have limited the interpretation of the data. In addition, because of the scanning protocol of OCT, we might have missed lesions between the scans of the images.

## Conclusion

Early AMD eyes with SDDs showed several notable features of RPE, and chorioretinal thinning was accompanied by features of RPE and sub-RPE deposits. Fellow eyes with neovascular AMD presented with relatively thicker retinas and choroids with greater proportions of RPE degeneration, and the occurrence of these relatively prominent degenerations of RPE before atrophic changes in the overall retina might be involved in the neovascularization process.

## Supplementary Information

**Additional file 1.** Supplementary table 1.

**Additional file 2.**Supplementary table 2.

**Additional file 3.**Supplementary table 3.

## Data Availability

The datasets used and/or analyzed during the current study available from the corresponding author on reasonable request. Unfortunately, the data is not publicly available due to local data protection laws.
