# Supplementary material for: Chorioretinal thickness and retinal pigment epithelial degeneration of fellow eyes in patients with unilateral neovascular age-related macular degeneration with subretinal drusenoid deposits
Source: BMC Ophthalmol. 2022 Jul 14;22:304. doi: 10.1186/s12886-022-02518-4 (PMC9284825; doi:10.1186/s12886-022-02518-4)
Supplement: Supplementary file 1 — Additional file 1. Supplementary table 1. [file 12886_2022_2518_MOESM1_ESM.docx]

Supplementary Table 1. Comparisons of retinal, ganglion–inner plexiform layer, and choroidal thickness values and the rate of retinal pigment epithelium degeneration among the non-neovascular age-related macular degeneration (AMD) with subretinal drusenoid deposit (SDD) group, neovascular AMD with SDD group, and control group

|  | SDD group | | Control group  (n = 47) | *P* value* |
| --- | --- | --- | --- | --- |
|  | Non-neovascular AMD  group (n = 47) | Neovascular AMD  group (n = 23) |  |  |
| Mean retinal thickness (µm) | 278.61 ± 13.96 **^a^** | 286.69 ± 15.02 **^b^** | 291.94 ± 14.05 **^b^** | <0.001 |
| Mean GCIPL thickness (µm) | 61.44 ± 4.63 **^a^** | 64.36 ± 4.21 **^b^** | 65.71 ± 6.56 **^b^** | 0.001 |
| Mean choroidal thickness (µm) | 133.59 ± 34.33 **^a^** | 156.11 ± 33.10 **^b^** | 175.16 ± 35.67 **^c^** | <0.001 |
| Nasal CT at 3000 µm (µm) | 77.00 ± 33.36 **^a^** | 107.35 ± 43.18 **^b^** | 140.91 ± 40.55 **^c^** | <0.001 |
| Nasal CT at 2250 µm (µm) | 91.64 ± 34.60 **^a^** | 119.09 ± 44.88 **^b^** | 156.87 ± 46.14 **^c^** | <0.001 |
| Nasal CT at 1500 µm (µm) | 110.96 ± 34.36 **^a^** | 138.13 ± 50.46 **^b^** | 172.17 ± 47.35 **^c^** | <0.001 |
| Nasal CT at 750 µm (µm) | 130.81 ± 38.65 **^a^** | 158.48 ± 49.32 **^b^** | 189.70 ± 42.57 **^c^** | <0.001 |
| Subfoveal CT (µm) | 142.98 ± 43.79 **^a^** | 171.7 ± 44.13 **^b^** | 207.32 ± 43.03 **^c^** | <0.001 |
| Temporal CT at 750 µm (µm) | 155.02 ± 46.82 **^a^** | 180.48 ± 42.53 **^b^** | 196.98 ± 39.53 **^b^** | <0.001 |
| Temporal CT at 1500 µm (µm) | 162.34 ± 48.86 **^a^** | 182.13 ± 36.28 **^b^** | 188.77 ± 41.90 **^b^** | 0.014 |
| Temporal CT at 2250 µm (µm) | 169.57 ± 54.30 **^a^** | 180.65 ± 36.17 **^a^** | 170.26 ± 38.68 **^a^** | 0.594 |
| Temporal CT at 3000 µm (µm) | 162.04 ± 53.42 **^a^** | 166.91 ± 35.27 **^a^** | 153.43 ± 34.71 **^a^** | 0.418 |
| Rate of RPE degeneration (%) | 38.3 | 65.2 | N/A | 0.034 |

SDD, subretinal drusenoid deposit; AMD, age-related macular degeneration; GCIPL, ganglion cell–inner plexiform layer; CT, choroidal thickness; RPE, retinal pigment epithelium

**P* value is based on the analysis of variance test. a, b, c: if followed by the same letter, groups do not differ significantly from one another according to the post-hoc analysis with Duncan’s test.
