# Supplementary material for: Chorioretinal thickness and retinal pigment epithelial degeneration of fellow eyes in patients with unilateral neovascular age-related macular degeneration with subretinal drusenoid deposits
Source: BMC Ophthalmol. 2022 Jul 14;22:304. doi: 10.1186/s12886-022-02518-4 (PMC9284825; doi:10.1186/s12886-022-02518-4)
Supplement: Supplementary file 2 — Additional file 2.Supplementary table 2. [file 12886_2022_2518_MOESM2_ESM.docx]

Supplementary Table 2. Comparisons of retinal, ganglion–inner plexiform layer, and choroidal thickness values and the rate of retinal pigment epithelium degeneration among the non-neovascular age-related macular degeneration with subretinal drusenoid deposit (SDD) group, type 3 macular neovascularization with SDD group, and control group

|  | SDD group | | Control group  (n = 47) | *P* value* |
| --- | --- | --- | --- | --- |
|  | Non-neovascular AMD  group (n = 47) | Type 3 MNV  group (n = 19) |  |  |
| Mean retinal thickness (µm) | 278.61 ± 13.96 **^a^** | 286.99 ± 15.96 **^b^** | 291.94 ± 14.05 **^b^** | <0.001 |
| Mean GCIPL thickness (µm) | 61.44 ± 4.63 **^a^** | 64.20 ± 4.01 **^b^** | 65.71 ± 6.56 **^b^** | 0.001 |
| Mean choroidal thickness (µm) | 133.59 ± 34.33 **^a^** | 151.84 ± 27.08 **^b^** | 175.16 ± 35.67 **^c^** | <0.001 |
| Nasal CT at 3000 µm (µm) | 77.00 ± 33.36 **^a^** | 96.79 ± 26.44 **^b^** | 140.91 ± 40.55 **^c^** | <0.001 |
| Nasal CT at 2250 µm (µm) | 91.64 ± 34.60 **^a^** | 111.63 ± 33.44 **^b^** | 156.87 ± 46.14 **^c^** | <0.001 |
| Nasal CT at 1500 µm (µm) | 110.96 ± 34.36 **^a^** | 130.00 ± 37.40 **^a^** | 172.17 ± 47.35 **^c^** | <0.001 |
| Nasal CT at 750 µm (µm) | 130.81 ± 38.65 **^a^** | 151.21 ± 38.63 **^b^** | 189.70 ± 42.57 **^c^** | <0.001 |
| Subfoveal CT (µm) | 142.98 ± 43.79 **^a^** | 166.84 ± 36.15 **^b^** | 207.32 ± 43.03 **^c^** | <0.001 |
| Temporal CT at 750 µm (µm) | 155.02 ± 46.82 **^a^** | 176.63 ± 38.37 **^b^** | 196.98 ± 39.53 **^b^** | <0.001 |
| Temporal CT at 1500 µm (µm) | 162.34 ± 48.86 **^a^** | 179.37 ± 34.38 **^ab^** | 188.77 ± 41.90 **^b^** | 0.016 |
| Temporal CT at 2250 µm (µm) | 169.57 ± 54.30 | 183.32 ± 33.91 | 170.26 ± 38.68 **^a^** | 0.501 |
| Temporal CT at 3000 µm (µm) | 162.04 ± 53.42 | 170.74 ± 31.77 | 153.43 ± 34.71 **^a^** | 0.309 |
| Rate of RPE degeneration (%) | 38.3 | 79.0 | N/A | 0.003 |

SDD, subretinal drusenoid deposit; AMD, age-related macular degeneration; MNV, macular neovascularization; GCIPL, ganglion cell–inner plexiform layer; CT, choroidal thickness; RPE, retinal pigment epithelium

**P* value is based on the analysis of variance test. a, b, c: if followed by the same letter, groups do not differ significantly from one another according to the post-hoc analysis with Duncan’s test.
