# Supplementary material for: Chorioretinal thickness and retinal pigment epithelial degeneration of fellow eyes in patients with unilateral neovascular age-related macular degeneration with subretinal drusenoid deposits
Source: BMC Ophthalmol. 2022 Jul 14;22:304. doi: 10.1186/s12886-022-02518-4 (PMC9284825; doi:10.1186/s12886-022-02518-4)
Supplement: Supplementary file 3 — Additional file 3.Supplementary table 3. [file 12886_2022_2518_MOESM3_ESM.docx]

Supplementary Table 3. Comparison of retinal, ganglion–inner plexiform layer, and choroidal thickness values between the non-neovascular age-related macular degeneration with and without retinal pigment epithelium degeneration groups and the control group

|  | Non-neovascular AMD | | | Control group  (n = 47) | *P* value* |
| --- | --- | --- | --- | --- | --- |
|  | RPE degeneration  group (n = 18) | No RPE degeneration  group (n = 29) | |  |  |
| Mean retinal thickness (µm) | 272.71 ± 11.18 **^a^** | 282.27 ± 14.43 **^b^** | | 291.94 ± 14.05 **^b^** | <0.001 |
| Mean GCIPL thickness (µm) | 58.34 ± 2.36 **^a^** | 63.36 ± 4.67 **^b^** | | 65.71 ± 6.56 **^b^** | <0.001 |
| Mean choroidal thickness (µm) | 108.29 ± 21.66 **^a^** | 149.29 ± 31.39 **^b^** | | 175.16 ± 35.67**^c^** | <0.001 |
| Nasal CT at 3000 µm (µm) | 63.72 ± 18.24 **^a^** | 85.24 ± 37.99 **^b^** | | 140.91 ± 40.55 **^c^** | <0.001 |
| Nasal CT at 2250 µm (µm) | 68.28 ± 15.93 **^a^** | 106.14 ± 35.27 | | 156.87 ± 46.14 **^c^** | <0.001 |
| Nasal CT at 1500 µm (µm) | 85.11 ± 20.81 **^a^** | 127.00 ± 31.31 | | 172.17 ± 47.35 **^c^** | <0.001 |
| Nasal CT at 750 µm (µm) | 105.78 ± 22.69 **^a^** | 146.34 ± 38.58 | | 189.70 ± 42.57 **^c^** | <0.001 |
| Subfoveal CT (µm) | 108.94 ± 38.25 **^a^** | 164.10 ± 32.48 **^b^** | | 207.32 ± 43.03 **^c^** | <0.001 |
| Temporal CT at 750 µm (µm) | 123.17 ± 34.34 **^a^** | 174.79 ± 42.76 **^b^** | | 196.98 ± 39.53 **^c^** | <0.001 |
| Temporal CT at 1500 µm (µm) | 132.17 ± 41.05 **^a^** | 181.07 ± 44.17 **^b^** | | 188.77 ± 41.90 **^b^** | <0.001 |
| Temporal CT at 2250 µm (µm) | 145.67 ± 46.00 **^a^** | 184.41 ± 54.45 **^b^** | | 170.26 ± 38.68 **^b^** | 0.021 |
| Temporal CT at 3000 µm (µm) | 141.94 ± 45.89 **^a^** | 174.52 ± 54.67 **^ab^** | 153.43 ± 34.71**^b^** | | 0.034 |

AMD, age-related macular degeneration; RPE, retinal pigment epithelium; GCIPL, ganglion cell–inner plexiform layer; CT, choroidal thickness

**P* value is based on the analysis of variance test. a, b, c: if followed by the same letter, groups do not differ significantly from one another according to the post-hoc analysis with Duncan’s test.
